# Quantitative systems toxicology (QST) reproduces species differences in PF‐04895162 liver safety due to combined mitochondrial and bile acid toxicity

**DOI:** 10.1002/prp2.523

**Published:** 2019-10-09

**Authors:** Grant Generaux, Vinal V. Lakhani, Yuching Yang, Sashi Nadanaciva, Luping Qiu, Keith Riccardi, Li Di, Brett A. Howell, Scott Q. Siler, Paul B. Watkins, Hugh A. Barton, Michael D. Aleo, Lisl K. M. Shoda

**Affiliations:** ^1^ DILIsym Services Inc. Research Triangle Park North Carolina; ^2^ Compound Safety Prediction Worldwide Medicinal Chemistry Pfizer Inc. Groton Connecticut; ^3^ Investigative Toxicology Drug Safety Research and Development Pfizer Inc. Groton Connecticut; ^4^ Pharmacokinetics, Dynamics and Metabolism Medicinal Sciences Pfizer Inc. Groton Connecticut; ^5^ UNC Eshelman School of Pharmacy University of North Carolina at Chapel Hill Chapel Hill North Carolina; ^6^ UNC Institute for Drug Safety Sciences University of North Carolina at Chapel Hill Chapel Hill North Carolina; ^7^ Translational Modeling and Simulation Biomedicine Design Pfizer, Inc. Groton Connecticut; ^8^ Present address: Division of Pharmacometrics Office of Clinical Pharmacology Office of Translational Sciences Center for Drug Evaluation and Research Food and Drug Administration Food and Drug Administration Silver Spring Maryland

**Keywords:** bile acid transporters, DILIsym, drug‐induced liver injury, mechanistic, mitochondria, PBPK, PF‐04895162, QSP, QST, species translation

## Abstract

Many compounds that appear promising in preclinical species, fail in human clinical trials due to safety concerns. The FDA has strongly encouraged the application of modeling in drug development to improve product safety. This study illustrates how DILIsym, a computational representation of liver injury, was able to reproduce species differences in liver toxicity due to PF‐04895162 (ICA‐105665). PF‐04895162, a drug in development for the treatment of epilepsy, was terminated after transaminase elevations were observed in healthy volunteers (NCT01691274). Liver safety concerns had not been raised in preclinical safety studies. DILIsym, which integrates in vitro data on mechanisms of hepatotoxicity with predicted in vivo liver exposure, reproduced clinical hepatotoxicity and the absence of hepatotoxicity observed in the rat. Simulated differences were multifactorial. Simulated liver exposure was greater in humans than rats. The simulated human hepatotoxicity was demonstrated to be due to the interaction between mitochondrial toxicity and bile acid transporter inhibition; elimination of either mechanism from the simulations abrogated injury. The bile acid contribution occurred despite the fact that the IC_50_ for bile salt export pump (BSEP) inhibition by PF‐04895162 was higher (311 µmol/L) than that has been generally thought to contribute to hepatotoxicity. Modeling even higher PF‐04895162 liver exposures than were measured in the rat safety studies aggravated mitochondrial toxicity but did not result in rat hepatotoxicity due to insufficient accumulation of cytotoxic bile acid species. This investigative study highlights the potential for combined in vitro and computational screening methods to identify latent hepatotoxic risks and paves the way for similar and prospective studies.

**Abbreviations**
ALP: alkaline phosphatase
ALT: alanine aminotransferase
BA: bile acid
BSEP: bile salt export pump
CDCA: chenodeoxycholic acid
DILI: drug‐induced liver injury
ETC: electron transport chain
FCCP: carbonyl cyanide‐p‐trifluoromethoxyphenylhydrazone
IC_50_: half‐maximal inhibitory concentrations
K_i_: inhibition constant
LCA: lithocholic acid
MRP3: multidrug resistance‐associated protein 3
NTCP: sodium/taurocholate cotransporting polypeptide
OCR: oxygen consumption rate
PBPK: physiologically based pharmacokinetic
QST: Quantitative Systems Toxicology
ROS: reactive oxygen species
ULN: upper limit of normal

## 1 INTRODUCTION

The FDA has developed the Advancing Regulatory Science Initiative (ARSI) as a strategic plan for improving the quality and efficiency of regulatory science. One aspect of this strategy is to develop computational methods and in silico modeling to improve product safety (https://www.fda.gov/science-research/advancing-regulatory-science/section-1-modernize-toxicology-enhance-product-safety-strategic-plan-regulatory-science). DILIsym software (DILIsym Services Inc, Research Triangle Park, NC) applies quantitative systems toxicology (QST) methods to model drug‐induced liver injury in silico^1^ and has been successfully used to improve the interpretation of clinical liver signals.^2^, ^3^, ^4^ Further, DILIsym has reproduced rat vs human species differences in toxicity.^5^, ^6^, ^7^ This study represents the first case characterized by transaminase elevations in humans but not rats that can be simulated by the combination of mitochondrial and bile acid toxicity.

PF‐04895162 (ICA‐105665, discovered by Icagen, Inc, Durham, NC) is a novel small molecule that was being developed for the treatment of epilepsy^8^ based on its ability to open Kv7.2/7.3 and Kv7.3/7.5 potassium channels.^9^

PF‐04895162 was evaluated in multiple preclinical studies. In a 7‐day rat toxicity study, dose‐dependent alanine aminotransferase (ALT) elevations, potentially indicative of liver toxicity, were observed. However, no histological evidence of liver injury was identified, and ALT elevations were not confirmed in a repeat 7‐day study. Further, 28 day and 6 month toxicity studies in rats were negative for transaminase elevations and liver toxicity, and toxicity studies up to 9 months duration in cynomolgus monkeys were also negative. Together, the preclinical studies did not identify liver as a target organ for safety concerns.^10^

In clinical studies, there was no substantial evidence of liver injury (ie, ALT > 3x ULN) in healthy subjects given single doses of PF‐04895162 up to 600 mg or multiple doses up to 200 mg twice daily (BID) for 7 days.^8^, ^11^ In another 7‐day study, one ALT elevation was noted among 12 subjects given 250‐mg BID or 300‐mg BID for 7 days (Pfizer data on file). Surprisingly, in a 14‐day study given 300‐mg PF‐04895162 BID, six of eight healthy subjects experienced ALT elevations, as high as 5x the upper limit of normal (ULN). This last study prompted discontinuation of the drug development program.^10^

PF‐04895162 has been examined as a potential case study for DILIsym in the evaluation of human but not rat transaminase elevations. DILIsym predicts hepatotoxicity by coupling *in vitro* data of hepatotoxic mechanisms with simulated predictions of *in vivo* exposure. Specifically, the *in vitro* data measure the interaction between PF‐04895162 and mitochondrial function as well as the compound's interaction with bile acid transporters. The *in vivo* exposure in human and rat subjects is predicted by a species‐specific physiologicallyl based pharmacokinetic (PBPK) model. Simulation results were found to reproduce clinical hepatotoxicity, without associated nonclinical hepatotoxicity. Additionally, the simulated human hepatotoxicity was found to require both PF‐04895162‐mediated mitochondrial dysfunction and bile acid transporter inhibition, as removal of either mechanism abrogated ALT elevations. Finally, simulations addressing alternate dosing protocols and uncertainty in liver exposure maintained consistency with clinical and preclinical observations. This case study supports the potential application of DILIsym to identify latent human liver safety liabilities.

## 2 MATERIALS AND METHODS

### 2.1 DILIsym overview

DILIsym is a QST‐based computational representation of drug‐induced liver injury (DILI)^12^, ^13^, ^14^, ^15^ that has been extensively used for evaluating the hepatotoxicity of drugs.^3^, ^4^ This software integrates multiple submodels (eg*,* hepatocyte life cycle, mitochondrial dysfunction and toxicity, bile acid disposition, and biomarker release) into a single simulation. DILIsym also allows for a PBPK representation of drug metabolism and disposition, which have been described previously.^6^, ^15^, ^16^, ^17^ The DILIsym software has been developed by the DILI‐sim Initiative, which is a public‐private partnership among scientists in academia, industry, and the US FDA.^14^ In this study, simulations were performed in the DILIsym v8A version of the software using parameter sets for the rat and human SimPops. Additional parameters specific to PF‐04895162 are described below.

### 2.2 In vitro assays for the representation of mechanisms of toxicity

To mechanistically simulate PF‐04895162‐mediated liver injury, in vitro data were collected to characterize the relationship between PF‐04895162 and mitochondrial toxicity, bile acid transporters, and oxidative stress. Data collection and derivation of parameter values based on the in vitro data have been described previously^3^, ^4^, ^5^, ^18^ and are described briefly below, with emphasis on the specifics of this project. PF‐04895162 was not observed to generate reactive oxygen species, and this mechanism has been omitted for brevity.

#### 2.2.1 Bile acid transporter input data

In DILIsym, the bile acid sub‐model captures the enterohepatic circulation of bile acids, including CDCA, CDCA‐amide, and LCA. There is an explicit representation of bile acid transporters such as NTCP, BSEP, MRP3, and MRP4. Half‐maximal inhibitory concentrations (IC_50_) were determined from standard vesicular transport assays for human BSEP, MRP3, and MRP4, as well as rat Bsep and Mrp3 (Solvo Biotechnology, Hungary) using taurocholate, β‐estradiol‐17‐β‐D‐glucuronide, and dehydroepiandrosterone sulfate as substrates, respectively. IC_50_ values were determined in Chinese Hamster Ovary (CHO) cells expressing human NTCP or rat Ntcp (Solvo Biotechnology, Hungary) using taurocholate as a substrate. Measured IC_50_ values were used directly in DILIsym. In cases where concentration‐dependent inhibition was measured that did not reach 50%, IC_50_ values were extrapolated. In the absence of inhibition constant (K_i_) data, which would define the mode of inhibition, the DILIsym standard practice is to represent bile acid inhibition as mixed, with an alpha value of 5.^4^ This alpha value provides a balance between competitive and noncompetitive inhibition.

#### 2.2.2 Mitochondrial dysfunction input data

DILIsym contains a mitochondria sub‐model, which represents cellular bioenergetics, including ATP levels. For the characterization of potential human mitochondrial toxicity, PF‐04895162 (3.7‐300 µmol/L) was cultured with primary human hepatocytes for 24 hours (Supporting Information 1 ‐ Methods). Compound effects on basal oxygen consumption rate (OCR) and respiratory reserve, as induced by the addition of FCCP (3 µmol/L), were measured using the Seahorse XF Analyzer (Seahorse Biosciences, Massachusetts) as previously detailed.^19^, ^20^ In similar fashion, the potential for rat mitochondrial toxicity was evaluated by evaluating basal OCR and respiratory reserve following culture of PF‐04895162 (1.23‐300 µmol/L) with primary rat hepatocytes for 1 or 24 hours (Supporting Information 1 ‐ Methods).

A companion software to DILIsym called MITOsym^®^ 21 was used to reproduce the measured Seahorse data, thus identifying parameter values that account for the activity of PF‐04895162 in vitro. MITOsym parameter values were then translated to in vivo DILIsym parameters using a conversion factor.^16^

### 2.3 PBPK modeling

For both rat and human, DILIsym PBPK sub‐models were used to represent PF‐04895162 pharmacokinetics consistent with that observed of in vivo plasma concentration profiles. The most parsimonious PBPK sub‐model possible was used to represent the individual species' pharmacokinetics. Rat required a slightly more complex PBPK sub‐model, due to the need to represent saturable gut absorption following oral administration. The rat PBPK sub‐model included compartments for blood, liver, muscle, gut, and other tissue. In the human simulations, a more simplified PBPK sub‐model was used, which included two compartments—blood and liver—with the remaining tissues aggregated into a general representation of systemic volume of distribution. For both sub‐models, the distribution of PF‐04895162 throughout the body was assumed to be perfusion‐limited.

### 2.4 Baseline simulated individual

The baseline simulated individual (in any species) represents a single (n = 1) normal healthy individual with roughly average anthropometric and biochemical characteristics. The baseline simulated rat and human were used for optimization of the PBPK sub‐models.

### 2.5 SimPops

SimPops are a collection of simulated individuals including parameter variability that reflects anthropometric and biochemical ranges. All human simulations were run with the Human_ROS_apop_mito_BA_v8A_1 (n = 285) SimPops. This group's parameter distribution reflects normal healthy volunteers (NHVs) with variability in body mass, mitochondrial function, caspase activation (apoptosis), bile acid transporter expression, and susceptibility to oxidative stress (ROS) (Supporting information 1, Table S1). Similarly, all rat simulations were run with the Rat_ROS_apop_mito_BA_v8A_11 (n = 294) SimPops, whose parameter distribution reflects a population of healthy rats with variability in the same physiological areas as the human SimPops (Supporting information 1, Table S2).

### 2.6 Human SimCohorts

SimCohorts are smaller collections of simulated individuals than SimPops. The Human_ROS_apap_mito_BA_v8A_1_Multi16_A (n = 16) SimCohort, which includes simulated individuals susceptible to mitochondrial dysfunction, bile acid accumulation, or oxidative stress, as well as relatively resistant individuals, was used for mechanistic investigations. In these investigations, PF‐04895162 was serially simulated with all mechanisms engaged, then in the absence of the mitochondrial dysfunction mechanism (only), and then in the absence of the bile acid toxicity mechanism (only). Loss of simulated individuals with ALT elevations in the absence of a particular mechanism was interpreted as implicating the absent mechanism as contributing to PF‐04895162 clinical toxicity.

### 2.7 Simulation protocols

For rat PBPK optimization, the following protocols were simulated: 1 mg/kg IV or 10, 30, or 100 mg/kg/day repeat daily dosing PO for up to 28 days. For human PBPK optimization, a 300‐mg single oral dose was simulated. The resulting PBPK sub‐model was then tested with 300‐mg BID repeat oral dosing. For toxicity simulations, simulated rats were administered 100 mg/kg/day PF‐04895162 PO for 28 days, while simulated humans received 300‐mg BID PO for 14 days, followed by a 14‐day washout period. Mechanistic investigations utilized the toxicity protocols (in the presence of all mechanisms, or all but one mechanism). Simulated humans also received 200‐mg BID PO for 7 days to reproduce an earlier trial that had no reported hepatotoxicity.^11^

### 2.8 Multiple linear regression analysis

In order to better understand which mechanistic parameters were significant factors in plasma ALT elevations, multiple linear regression analysis was performed using the R Language and Environment for Statistical Computing (R Project, https://www.r-project.org/). Because the magnitude of different parameter values included within the SimPops span a large range, each parameter's values were normalized by dividing that parameter's value by its maximum parameter value within the SimPops in order to result in a range for all parameter values of 0‐1.

## 3 RESULTS

### 3.1 In vitro experimental data

#### 3.1.1 Bile acid transporter data

**Figure 1:**
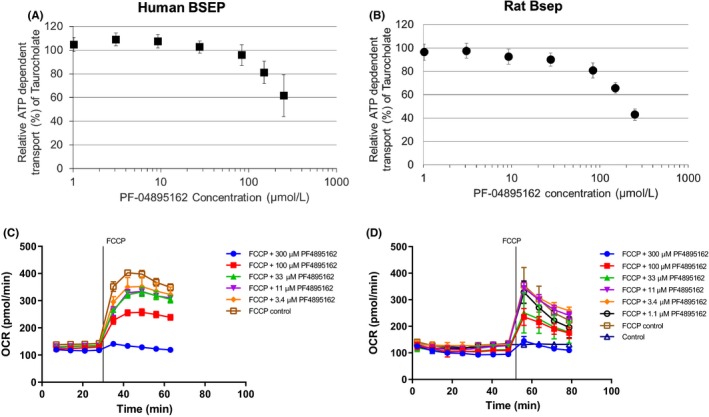
Examples of in vitro data collected to quantitatively characterize the relationship between PF‐04895162 and mechanisms of toxicity. (A) PF‐04895162 interaction with human BSEP in vesicular transport assays (SB‐BSEP‐HEK293), using 0.2 µmol/L taurocholate as the probe substrate. PF‐04895162 inhibited BSEP‐mediated taurocholate transport by 38% at 250 µmol/L (maximum tested concentration). (B) PF‐04895162 interaction with rat Bsep in vesicular transport assays (SB‐ratBsep‐HEK293), using 2 µmol/L taurocholate as the probe substrate. The calculated IC_50_ value was 229.6 µmol/L. (C) Oxygen consumption rate (OCR) following 24‐hour incubation of primary human hepatocytes with PF‐04895162. Basal OCR was measured in a Seahorse XF Analyzer for 40 minutes, followed by injection of FCCP (3 µmol/L) and measurement of the increase in OCR as an indicator of spare respiratory capacity. Two independent studies were conducted, with similar results. (D) OCR following 1‐hour incubation of rat primary hepatocytes with PF‐04895162. Basal OCR and spare respiratory capacity were measured using a Seahorse XF Analyzer. Two independent studies were conducted, with similar results. Studies using 24‐hour incubation with PF‐04895162 were also conducted, with similar results

Concentration‐dependent interactions were observed between PF‐04895162 and both human and rat bile acid transporters (eg, Figure 1A and B). More specifically, PF‐04895162 inhibited human MRP4 bile acid efflux transporter, with an IC_50_ value of 121 μmol/L (Supporting Information 2 Figure S1A). An IC_50_ value could not be directly calculated from the measured BSEP or MRP3 bile acid efflux transporter data due to insufficient inhibition. However, small, apparently dose‐dependent decreases were noted at the highest tested concentrations of PF‐04895162. The extrapolated IC_50_ values for BSEP and MRP3 were 311 and 519 μmol/L (Supporting Information Figure S1B and C, respectively). Similarly, an IC_50_ value could not be directly calculated from the measured NTCP data, although dose‐dependent inhibition was noted at the highest tested concentrations. The extrapolated NTCP IC_50_ was 1535 μmol/L (Supporting Information Figure S1D).

PF‐04895162 was also an inhibitor of rat Mrp3 and Bsep bile acid efflux transporters, with measured IC_50_ values of 256 and 229.6 μmol/L, respectively (Supporting Information Figure S2A and B). PF‐04895162 inhibited rat bile acid uptake via Ntcp, with an IC_50_ value of 198 μmol/L (Supporting Information Figure S2C).

#### 3.1.2 Mitochondrial dysfunction data

Incubation of human hepatocytes with PF‐04895162 for 24 hours had minimal effect on basal respiration, as measured by OCR. However, PF‐04895162 dose‐dependently reduced hepatocyte spare respiratory capacity, as measured by abrogation of the FCCP‐mediated increase in OCR (Figure 1C). Similarly, incubation of rat hepatocytes with PF‐04895162 for 1 or 24 hours had minimal effect on basal respiration while dose‐dependently reducing spare respiratory capacity (Figure 1D). Based on these data, PF‐04895162 was presumed to act as a mild electron transport chain (ETC) inhibitor, where inhibitory effects were unmasked by the addition of FCCP.

The in vitro data describe the relationship between the nominal media concentration of PF‐04895162 and hepatocyte respiration. To more accurately describe the concentration‐effect relationship at the site of action (ie*,* intracellularly), cell‐associated PF‐04895162 was measured. PF‐04895162 concentrations were higher in the cell‐associated fraction relative to the measured media concentration and to the protocol‐specified nominal media concentration (Table S3). The cell‐associated concentration was used to identify parameter values that characterize the observed relationship between PF‐04895162 and mitochondrial dysfunction as described above.

### 3.2 Parameter values for toxicity mechanisms

#### 3.2.1 PF‐04895162 parameter values for bile acid transporter inhibition

DILIsym uses IC_50_ values directly for canalicular (ie, BSEP, Bsep), basolateral efflux (ie, MRP3/4, Mrp3/4), and basolateral influx (ie, NTCP, Ntcp). Where IC_50_ values could be directly calculated from the measured data, those values were used. Where the degree of inhibition was insufficient for direct calculation, but a lesser degree of dose‐dependent inhibition was observed, the IC_50_ value was extrapolated from the data (Figures S1A‐D and S2A‐C, for human and rat parameter values, respectively). In the case of basolateral efflux, the lower of the measured IC_50_ values was applied in order to provide a more conservative perspective on liver safety. As described in the Methods, the mode of inhibition was specified as mixed inhibition with an alpha value of 5 representing a balance between competitive and noncompetitive inhibition per standard DILIsym practice^4^ (Table S4).

#### 3.2.2 PF‐04895162 parameter values for ETC inhibition

The experimental respiration data showing dose‐dependent inhibition of the FCCP‐mediated increase in human and rat hepatocyte respiration by PF‐04895162 were reproduced in MITOsym using the ETC inhibition mechanism 1 (Supporting Information Figures S3 and S4, respectively). The resultant DILIsym parameter values are listed in Table S4.

### 3.3 Rat and human PBPK sub‐model optimization

**Figure 2:**
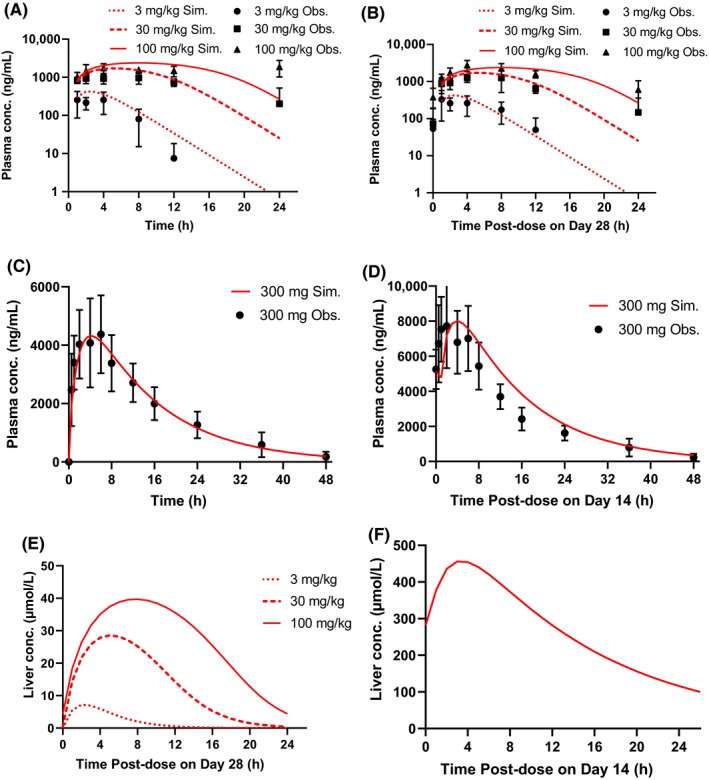
Comparisons of PBPK sub‐model simulations against measured data. The rat PBPK sub‐model was simultaneously optimized against multiple dosing protocols. (A) Simulated rat plasma PF‐04895162 (ng/mL) following single oral administration of 3, 30, or 100 mg/kg was compared with measured data for the same protocols. (B) Simulated rat plasma PF‐04895162 (ng/mL) for study day 28 following repeat daily dosing of 3, 30, or 100 mg/kg was compared with measured data for the same protocols. (C) Simulated human plasma PF‐04895162 (ng/mL) following optimization against data for a single 300‐mg po dose. (D) Simulated human plasma PF‐04895162 (ng/mL) from day 14, following 300‐mg BID dosing, as compared with data. The human repeat dosing simulation result did not require further changes to the human PBPK sub‐model optimized to a single 300‐mg dose. (E) Simulated rat liver concentrations on day 28 following administration of 100 mg/kg/day. (F) Simulated human liver concentrations on day 14 following administration of 300‐mg BID for 14 days

The rat PBPK sub‐model was optimized to experimental data on plasma concentrations of PF‐04895162 following a single administration of 1 mg/kg IV, or repeat dosing of 10, 30, or 100 mg/kg/day PO in repeat dosing out to 28 days. Experimental data were used either directly or as constraints on the parameter space during optimization. The resultant set of parameter values (Table S5) yielded plasma PF‐04895162 concentrations and dynamics that were consistent with measured data (Figure 2A and B and Figures S5).

The human PBPK sub‐model was optimized to experimental data on plasma concentrations following a single dose of 300‐mg PF‐04895162. The resultant set of parameter values (Table S6) yielded plasma PF‐04895162 concentrations and dynamics that were consistent with the measured data after both single dose and 14‐day repeat dose administration (Figure 2C and D).

The average PBPK‐predicted rat and human liver concentrations for PF‐04895162 are shown in Figure 2E and F, respectively. The simulated rat liver to blood ratio was compared against in vivo quantitative whole‐body autoradiography (QWBA) data (Pfizer data on file) to confirm that simulated liver concentrations were consistent with measured data. Of course, no corresponding in vivo liver data were available for humans. In the absence of these data, liver concentrations were compared with in vitro data on intracellular accumulation of PF‐04895162 in primary human hepatocytes (Table S3).

### 3.4 Hepatotoxicity simulations

#### 3.4.1 PF‐04895162 induces ALT elevations in human but not in rat SimPops

**Figure 3:**
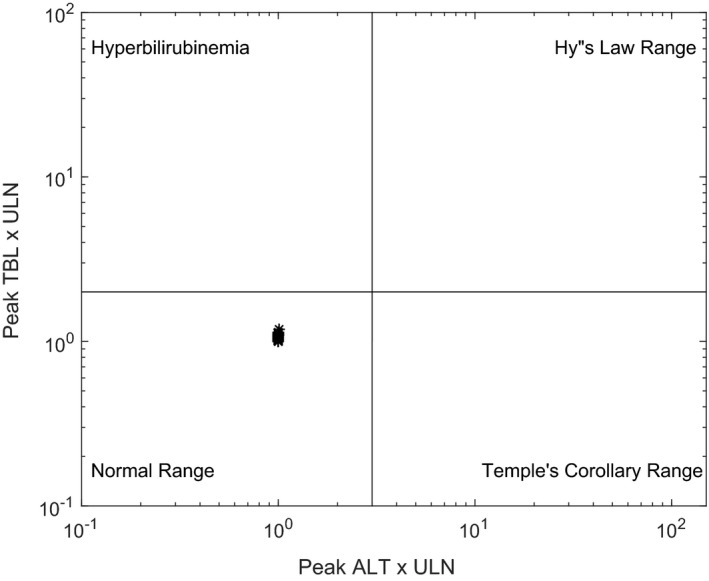
Simulation of PF‐04895162 (100 mg/kg/day for 28 days) in rat SimPops (n = 294) does not result in hepatotoxicity. Evaluation of drug‐induced serious hepatotoxicity (eDISH) plot for rat SimPops results, illustrating peak ALT (x‐axis) vs peak total bilirubin (y‐axis) for each individual. Each star represents peak ALT and total bilirubin for an individual rat. Vertical lines correspond to 3x ULN for ALT and 2x ULN for total bilirubin

**Figure 4:**
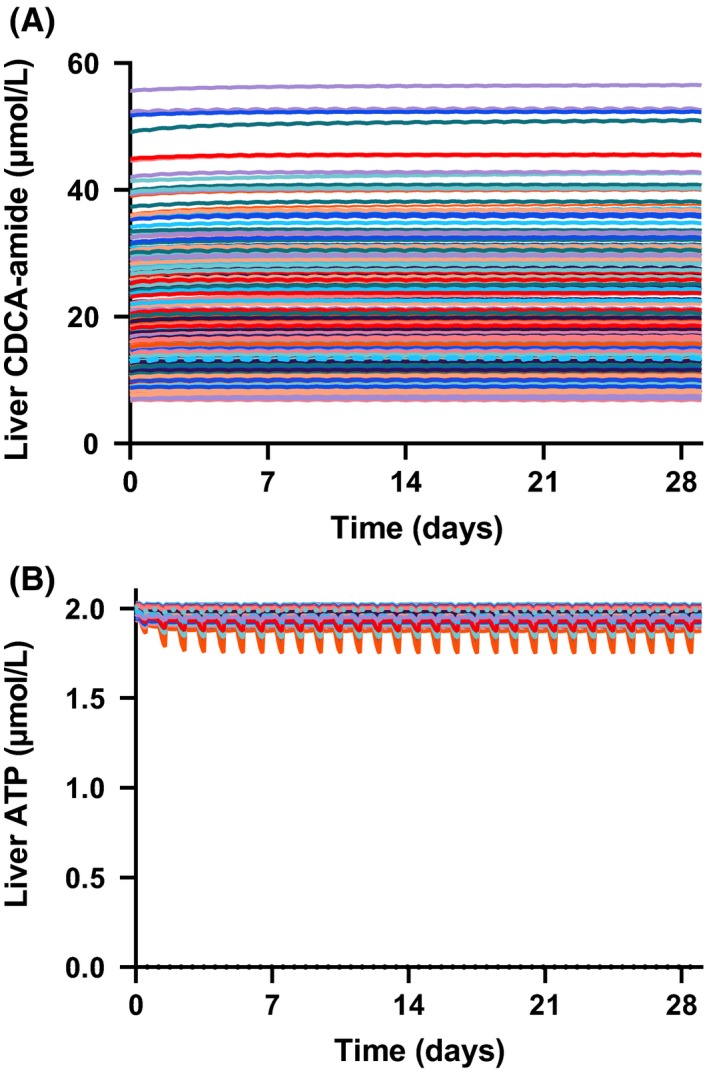
Subclinical simulated indicators of mechanisms of toxicity in rat SimPops. (A) Liver CDCA‐amide across the 28‐day dosing period (100 mg/kg/day). Each line represents an individual rat. (B) Liver average ATP across the 28‐day dosing period (100 mg/kg/day)

Simulations incorporating PBPK sub‐models and the two putative mechanisms of toxicity were conducted in human and rat SimPops, using species‐specific protocols as specified in the Methods section. Consistent with preclinical results, the simulation in rats did not show evidence of hepatotoxicity due to PF‐04895162, as indicated by the eDISH plot (Figure 3). The eDISH plot is a typical way by which liver safety is evaluated in clinical trials.^22^ Further mechanistic investigation into the simulated dynamics of bile acids and ATP showed that levels of the hepatotoxic bile acid CDCA‐amide were largely unaffected and only modest decreases were simulated for liver ATP levels (Figure 4A and B).

**Figure 5:**
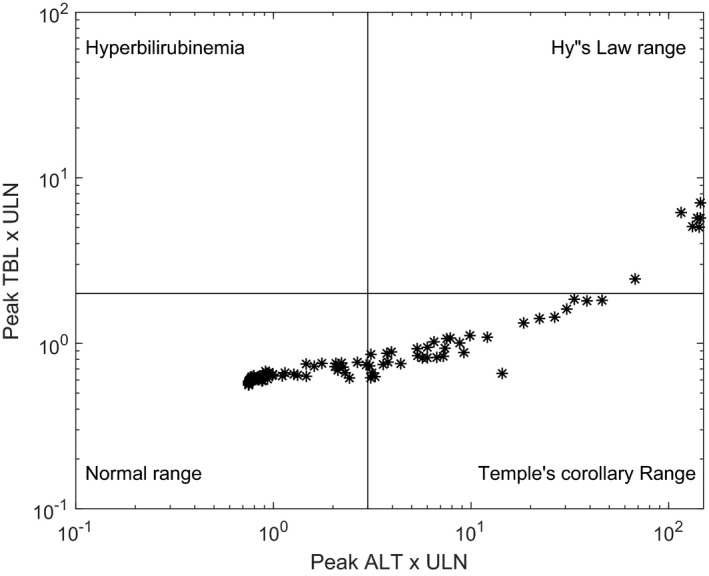
Simulation of PF‐04895162 (300‐mg BID for 14 days) in human SimPops (n = 285) results in hepatotoxicity. Each star represents peak ALT and total bilirubin for an individual human subject in the eDISH plot

In contrast to rat simulation results, human simulations with PF‐04895162 did show evidence of hepatotoxicity, as seen in the eDISH plot (Figure 5). The human SimPops simulation predicted ALT > 1x ULN in 59/285 (21%), ALT > 5x ULN in 32/285 (11%), and ALT > 10x ULN in 18/285 (6%) of simulated individuals. Bilirubin elevations in excess of 2x ULN were predicted in 9/285 simulated individuals. By comparison, the observed clinical results showed ALT > 1x ULN in 6/8 (75%), ALT > 5x ULN in 1/8 (12%), and ALT > 10x ULN in 0/8 (0%) subjects. The clinical results also included two subjects with total bilirubin elevations between 1 and 1.5x ULN.^10^ Thus, while the species difference is broadly in agreement with the measured data, simulated human ALT elevations were less frequent and sometimes more severe than those observed clinically in the small Phase 1 multidose trial with eight human subjects (Table 1).

**Table 1:** Comparison of PF‐04895162‐induced ALT elevations in human SimPops relative to clinical data

	ALT > 1x ULN	ALT > 5x ULN	ALT > 10x ULN
Simulated	59/285 (21%)	32/285 (11%)	18/285 (6%)
Observed	6/8 (75%)	1/8 (12.5%)	0/8 (0%)

Simulations predicted nine individuals (9/285, 3%) with severe liver injury, qualifying as Hy's Law cases (ie*,* ALT > 3x ULN, total bilirubin > 2x ULN). This particular simulation result stands in stark contrast to the clinical data in which the most severe response was a Grade 3 ALT elevation (5‐10x ULN), absent bilirubin elevation. Multiple regression analysis of parameter variability included within the SimPops suggested that body weight, as a covariate for PF‐04895162 exposure, as well as decreased capacity for bile acid canalicular efflux were the most significant factors in elevated plasma ALT levels (Table 2). Consistent with this analysis, the nine Hy's Law individuals were generally on the low end of the parameter spectrum with respect to body mass and canalicular efflux capacity. Overall, the analysis suggests multifactorial susceptibility in this small number of simulated patients.

**Table 2:** Results from multiple linear regression analysis of maximum ALT elevations in human SimPops

Parameter name in DILIsym^a^	Parameter description	*P* value
Body_mass	Body mass	0.000032
BA_canal_Vmax	Bulk bile acid canalicular transport Vmax	0.00078
canal_reg_scale	Canalicular transporter regulation exponent	0.00014

a Only parameters meeting the statistical threshold of *P* < .001 are listed. Body mass is related to ADME (exposure). Bulk bile acid canalicular transport *V*_max_ and canalicular transporter regulation exponent are related to canalicular bile acid efflux.

**Figure 6:**
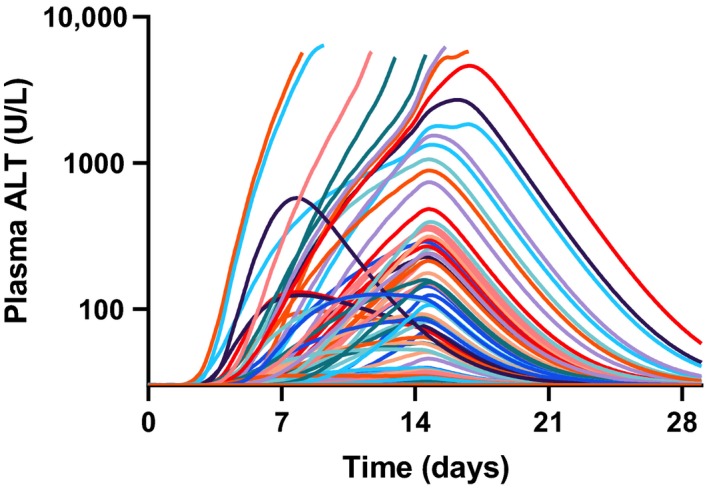
Simulated time courses for ALT in human SimPops treated with PF‐04895162 (300‐mg BID for 14 days and followed for an additional 14 days). Each line represents an individual subject

**Figure 7:**
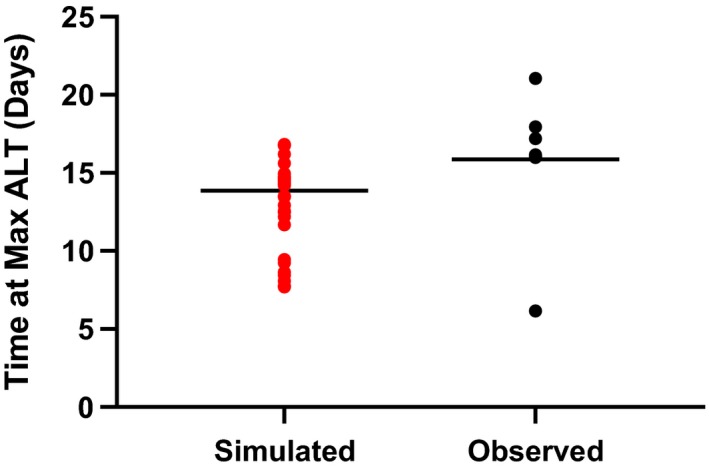
Comparison of time to peak ALT in human SimPops vs measured clinical data. Simulated individuals are represented in red. Clinical measurements for subjects in the Phase I study are represented in black. The median value is indicated by a line

The timing of the peak serum ALT elevation in the DILIsym simulation was in good agreement with the observed clinical data (Figure 6). Clinically, most patients saw peak ALT levels occurring between days 14 and 19; one individual had a peak ALT around day 6.^10^ The human SimPops simulation showed a median time to peak ALT value of 14.2 days, with an overall range of 7.7‐16.8 days. Clinically, the observed median time to peak ALT value was 16.0 days and ranged between 6.2 and 21.0 days (Figure 7).

**Figure 8:**
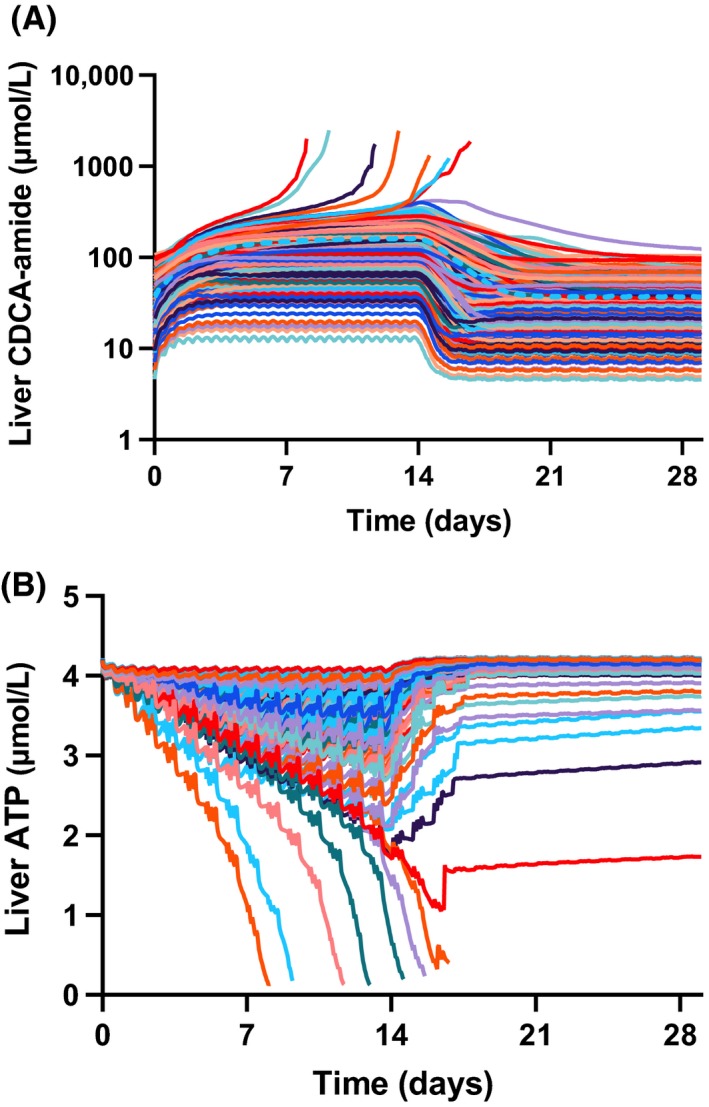
Subclinical simulated indicators of mechanisms of toxicity in human SimPops. (A) Liver CDCA‐amide across the 14‐day dosing period (300‐mg BID), with 14‐day follow‐up. Each line represents an individual subject. (B) Liver average ATP across the 14‐day dosing period (300‐mg BID), with 14‐day follow‐up

Further mechanistic investigation into the simulated dynamics of ATP and bile acids in the human simulations showed more substantial decreases in liver ATP levels compared to those observed in rat, as well as significant increases in the hepatic levels of the hepatotoxic bile acid CDCA‐amide (Figure 8A and B).

To investigate which mechanism was primarily responsible for the hepatotoxicity observed in the human SimPops simulation, a smaller subset of 16 simulated patients (SimCohort) was run with either ETC or bile acid transporter inhibition turned off (Table 3). Interestingly, when either PF‐04895162‐mediated ETC inhibition or transporter inhibition was removed as a mechanism of toxicity, none of the simulated individuals had ALT elevations. The results suggest that PF‐04895162‐mediated BSEP inhibition could contribute to the observed liver signals in the presence of ETC inhibition, even with the relatively low potency of an IC_50_ of 311 μmol/L. Together, the simulation results argue for the synergistic activity between BSEP and ETC inhibition.

**Table 3:** Investigations into mechanistic drivers of toxicity in SimCohorts^a^

Simulations	Mechanisms on	Mechanisms off	ALT Elevations > 3x ULN
300‐mg po BID for 14 days in Multi16^b^	ETCi, BAi	—	8/16
	ETCi	BAi	0/16
	BAi	ETCi	0/16

a Because this SimCohorts includes individuals selected for sensitivity to different mechanisms of toxicity, they are useful to screen for sensitivity to different mechanisms but not to evaluate frequency of hepatotoxicity

b Multi16 is the Human_ROS_apap_mito_BA_v8A_1_Multi16_A (n = 16) SimCohort

Even with underlying mechanisms demonstrated in the human SimPops, it seemed important to consider the possible contribution of exposure. The predicted human liver exposure exceeded the predicted rat liver exposure (Figure 2E and F). Although liver exposure for each species was grounded in species‐specific data, the nature of the data differed (ie*, *in vivo Quantitative Whole‐Body Autoradiography or QWBA data for rats, in vitro cell‐associated data for human hepatocytes). Since data were also available for intracellular accumulation of PF‐04895162 in rat hepatocytes, an alternate rat PBPK sub‐model was developed that maintained consistency with measured plasma levels but with higher liver accumulation consistent with the in vitro data (Figure S6A and B). Despite the higher liver concentration, PF‐04895162 simulation in the rat remained relatively clean, with peak ALT in one rat exceeding 40 U/L (Figure S7), due to limited accumulation of cytotoxic bile acid species despite greater mitochondrial toxicity (Figure S8A and B). Surprisingly, results also demonstrated mild bilirubin elevations, but these were due to the effect of reduced ATP on bilirubin transporter function and are thus not reflective of liver injury. Thus, even with liver concentrations aligned with in vitro data but vastly in excess of those measured in vivo, simulated species differences were maintained.

In addition to the contribution of exposure, simulations were conducted to explore the robustness of the predictions to protocol variation. Specifically, the human SimPops was tested with 200‐mg BID, administered over 7 days. A small clinical trial applying this protocol in 24 NHVs did not include ALT elevations exceeding 3x ULN^11^ The human SimPops results applying this same protocol were largely clean in 283/285 individuals, excepting two individuals with ALT elevations > 3x ULN but no bilirubin elevation (Figure S9). The simulation results confirm that simulated Kv7 replicates the apparent dose‐dependency in ALT elevations observed in clinical trials. Ideally, one might have hoped for a completely clean simulation result, but the small number of individuals in the clinical trial raises the possibility that full population susceptibility was not represented. The two simulated individuals with ALT elevations had low body weights (50 and 53 kg), with attendant higher exposure which likely contributed to their susceptibility. Additional dose exploration indicated that simulation of 170‐mg BID, 7‐day exposure, yielded no ALT elevations > 3x ULN (results not shown). Together, the investigation of (a) higher liver exposure in rats, with no predicted hepatotoxicity consistent with preclinical data, and (b) lower dosing in humans, with loss of predicted hepatotoxicity, consistent with clinical data support the overall contention that the integration of exposure and mechanisms of toxicity reproduce species differences in hepatotoxicity.

## 4 DISCUSSION

The simulations reproduced the species difference in hepatotoxicity, including the delayed presentation in ALT elevations. Mechanistic analyses demonstrated that the hepatocyte injury (and resulting ALT elevations) could be accounted for by the synergistic action of PF‐04895162 on mitochondrial function and bile acid transporters. More specifically, PF‐04895162 inhibition of bile acid transporters drove accumulation of intracellular bile acids, which induced mitochondrial toxicity.^23^ Bile acid‐mediated mitochondrial toxicity combined with PF‐04895162‐mediated ETC inhibition disrupted mitochondrial function to a greater degree than either mechanism individually. Dual contribution of compromised mitochondrial function resulted in cell death and associated ALT elevations in simulated humans but not rats. The species difference was multifactorial. In part, the species difference could be attributed to lower simulated liver accumulation of PF‐04895162 in rats compared to humans. In rats, the simulated liver accumulation could be guided in part by in vivo data from QWBA studies which were not available in human. Simulated liver accumulation in humans was informed by the available human data (ie*, *in vitro accumulation of PF‐04895162 in primary human hepatocytes, Table S3). It remains possible that actual human liver accumulation was less than the predicted liver concentration, but this possibility and its extent are unknown. By contrast, we could consider higher rat liver concentrations as informed by in vitro data rather than as indicated by the in vivo data. Despite greater liver exposure, simulated rats remained insensitive to PF‐04895162‐mediated hepatotoxicity due to modest engagement of toxicity mechanisms and perhaps also to the species‐specific accumulation of less toxic bile acid species.^24^

In simulations, delayed ALT presentation reflected the time required for PF‐04895162‐mediated bile acid transporter inhibition to lead to sufficient bile acid accumulation for synergistic toxicity (Figure 8A and B). As has been previously reported, bile acid accumulation to cytotoxic levels may proceed over weeks to months, with attendant delays in ALT elevations.^7^ In the case of PF‐04895162, the delay was shortened by combination with mitochondrial toxicity, a reported risk factor for DILI when combined with BSEP inhibition.^25^ Given these results, it is tempting to speculate on the underlying mechanisms for flupirtine hepatotoxicity beyond genetic risk factors.^26^ Flupirtine is also a Kv7 potassium channel opener. It has been characterized as a BSEP inhibitor, with an IC_50_ value of 35.5 µmol/L^27^, ^28^ like PF‐04895162, also has mitochondrial inhibitory activity (~14 µmol/L, Aleo unpublished results). While more potent than PF‐04895162 as a BSEP inhibitor, it has weaker mitochondrial inhibitory effects, which may explain the extended timing needed to generate hepatotoxicity.^29^ Interruption of systemic bile acid homeostasis is observed as several individual bile acids were markedly elevated in flupirtine‐induced hepatotoxicity, including glycochenodeoxycholic acid, taurochenodeoxycholic acid, and taurocholic acid^26^ similar to that observed with PF‐04895162 before significant transaminase or total bile acid elevations were observed.^10^ In both cases, this was not simply the result of overt cholestasis since alkaline phosphatase (ALP) was normal. Further in vitro assay data and full DILIsym modeling would be required to determine if this hypothesis is quantitatively supported. Lastly, it is noteworthy that the mechanisms represented for PF‐04895162 were sufficient to account for a roughly 2‐week delay in ALT elevations absent a simulated adaptive immune response. Thus, PF‐04895162 provides another example of delayed toxicity due to intrinsic toxicity (vs adaptive immune‐mediated cytotoxicity).

The mechanistic underpinnings of the simulation results are supported by a previously published analysis of PF‐04895162.^10^ In vitro data identified interactions of PF‐04895162 with human mitochondrial respiration and bile acid transporters. Furthermore, a new biomarker analysis of residual plasma PK samples showed evidence of disrupted bile acid homeostasis.^10^ Notably, the in vitro data presented herein reflect a separate analysis of human and rat bile acid transporter inhibition, as well as mitochondrial dysfunction; the separate analysis was required to generate parallel human and rat data sets to inform the simulation parameter values.

While the simulations successfully reproduced species differences in toxicity and delayed presentation, there were discrepancies between simulated and observed outcomes, including frequency and magnitude of ALT elevations (Table 1). Comparing frequency against a small group of treated patients (n = 8) does carry some uncertainty, particularly because earlier clinical trials of lesser dose or duration had no or minimal liver safety signals.^10^ Nevertheless, the discrepancy in frequency could reflect the presence of an additional mechanism of toxicity. Within DILIsym, other mechanisms of toxicity include production of reactive metabolites, oxidative stress, lipotoxicity, and inflammation (eg, TNF‐α‐mediated cell death). Preclinical assays were negative for the generation of reactive metabolites or oxidative stress (Pfizer data on file). Drug‐induced lipotoxicity or inflammation was not included in standard screens but was also not expected to play a role based on the pharmacodynamic profile. Although these DILI mechanisms cannot be excluded, they are not likely candidates particularly in healthy subjects. Perhaps the more likely explanation is that the high clinical frequency reflects an additional mechanism of drug‐induced toxicity (eg, ER stress), which is not currently included in DILIsym. Simulations also overpredicted the magnitude of response. While the overprediction could be attributed to the small clinical study, it remains possible that administration of PF‐04895162 at 300‐mg BID to more individuals could have resulted in more severe ALT elevations. Another potential explanation is that the magnitude of simulated ALT elevations is due to the absence of an adaptive mechanism(s). For example, mitochondria can adapt to stress via mitochondrial biogenesis.^30^ The production of additional mitochondria might plausibly relieve the direct bioenergetics stress imposed by PF‐04895162, thereby reducing the severity of injury or possibly even eliminating the synergistic toxicity. Mitochondrial biogenesis has recently been added to DILIsym but is not included by default in simulations due to the lack of quantitative data available to support the selection of mitochondrial biogenesis parameter values. Finally, simulations also overpredict the magnitude of response due to the absence of simulated clinical monitoring. Simulated treatment continues regardless of the signs or symptoms that would be associated with liver injury, until hepatocyte loss exceeds 80% when simulations are terminated.

Research on PF‐04895162, after its discontinuation, was conducted in part to understand the potential for screening assays to detect liver liabilities. A compelling case has been built for the use of bile acid transporter inhibition assays in this application^27^, ^28^; although, the IC_50_ values for PF‐04895162 (all >100 µmol/L) greatly exceeded a recent consensus threshold for concern (25 µmol/L).^24^ A case has also been made for the use of bile acid transporter data in combination with mitochondrial function for the identification of compounds with liver liabilities.^25^ The results reported herein support and extend the use of these in vitro data, which illustrate how a computational approach can unmask a synergistic effect, easily overlooked when factors are considered separately. These results also provide a mechanistic explanation for the observed species difference in hepatotoxicity, supporting the potential application of this approach to prospectively identify latent liver liabilities before a compound is introduced into the clinic. Further case studies are warranted.

In conclusion, this study demonstrates the ability of a computational approach to reproduce species differences in hepatotoxicity, using PK data and in vitro data on mechanisms of toxicity, even though the in vitro data suggest an intrinsically weak engagement of these mechanisms. Similar case studies are warranted to further test the system and could potentially better characterize the strengths and weaknesses of rat liver toxicity studies from a QST mechanism‐based perspective. Ultimately, they would be useful to determine the application of DILIsym to improve the likelihood of identifying latent DILI liabilities.

## DISCLOSURES

The authors report that this research did not receive external public or private foundation funding. The study was sponsored by Pfizer. Authors are former or current employees of Pfizer or DSS or an advisory board member of DSS (PBW) and may continue to hold stock or other equity positions with the companies.
