# Supplementary material for: Quantitative systems toxicology (QST) reproduces species differences in PF‐04895162 liver safety due to combined mitochondrial and bile acid toxicity
Source: Pharmacol Res Perspect. 2019 Oct 9;7(6):e00523. doi: 10.1002/prp2.523 (PMC6785660; doi:10.1002/prp2.523)
Supplement: Supplementary file 1 [file PRP2-7-e00523-s001.pdf]

Supporting Information 1

Supporting Information Materials and Methods

Evaluation of PF-04895162 on mitochondrial respiration in human hepatocytes

Human hepatocytes (lot #4019 from Invitrogen) were seeded at 7,000 cells/well in an XF96-well plate in cell culture medium (Day 1). The cell culture medium was William's E medium (A1217601 Fisher Scientific) containing Thawing Medium supplement (CM3000, Fisher Scientific). Matrigel (351236, Corning) was added on top of the cells the next day (Day 2). The cells were dosed with the test compounds in cell culture medium 24 h later (Day 3). Twenty-four hours after the cells were dosed, the cell culture medium with test compounds was replaced with Krebs Henseleit buffer, pH 7.4 supplemented with 5 mM glucose and 6 mM glutamine (Day 4). Basal oxygen consumption rates (OCR) (3-30mins) and respiratory capacity (30-62mins) of the cells were determined on an XF96 (Seahorse Bioscience) platform.

Evaluation of PF-04895162 on mitochondrial respiration in rat hepatocytes

Fresh rat hepatocytes were obtained from Lonza (RSFS1M, Lonza) and seeded at 3500 cells/well in an XF96-well in William' s E medium (A1217601 Fisher Scientific) containing Thawing Medium Supplement (CM3000, Fisher Scientific) on day 1. The next day, 100ul of 0.25 mg/ml of Matrigel (351236, Corning) diluted in William's medium with Plating Medium Supplement (CM4000, Fisher Scientific) were added on top of the cells. 24 hrs after adding Matrigel (Day 3) , cells were dosed with test compounds in William 'E medium with Plating Medium Supplement for 1 hr or 24 hrs. Cells were replenished with Krebs Henseleit buffer, pH 7.4 supplemented with 5 mM glucose and 6 mM glutamine (Day 4) before the oxygen consumption rates were measured in the basal level (1-30min), under the treatment of compounds (30-50 min) and respiratory capacity of the cells (50-80mins) on an XF96 (Seahorse Bioscience) platform.

SimPops used in toxicity simulations

TABLE S1. Human SimPops: normal healthy volunteer (NHV) SimPops (Human_ROS_apop_mito_BA_v8A_1, n=285)

Data used to Define Parameter Distributions (if applicable)		
**Parameter Symbol in DILIsym**	**Parameter Name in DILIsym**	**Data Source for Distribution**
ATP_decr_necrosis_Vmax	ATP decrement necrosis Vmax	Assumed standard deviation of ±20% and parameter range of 2.5 times the S.D. and validated with outcome data
Body_mass	Body Mass	Parameter range from NHANES III (human data)
GSH_pre_trans_Vmax	GSH precursor transport Vmax	Parameter range derived from (Allen et al., 2001)
GSHo	GSH basal level	Parameter range from (Lee et al., 2007; Nagasaka et al., 2009)
HGF_prod_LSEC_Vmax	Maximum LSEC HGF production rate per liver LSEC	Assumed standard deviation of ±20% and parameter range of 2.5 times the S.D. and validated with outcome data
HGF_regen_Vmax	HGF mediated regeneration Vmax	Assumed standard deviation of ±20% and parameter range of 2.5 times the S.D. and validated with outcome data
RNS_ROS_ATP_inhib_Vmax	RNS/ROS ATP inhibition Vmax	Parameter range derived from (Shon and Nam, 2002)
RNS_ROS_cl_Vmax	Liver RNS/ROS baseline clearance Vmax	Assumed standard deviation of ±20% and parameter range of 2.5 times the S.D. and validated with outcome data
Basal_Stdzd_MitoETC_Flux	Basal value of mito ETC flux	Parameter range from healthy volunteer data (Pérez-Carreras et al., 2003)
Resp_Reserve_Scalar	Scaling coefficient representing reserve mitochondria function	Parameter range from healthy volunteer data (Pérez-Carreras et al., 2003)
CAS_apop_scale	Caspase-mediated apoptosis scaling constant	Parameter range derived from (Bantel et al., 2001)
BA_uptake_Vmax	Bulk bile acid uptake Vmax	All transporters were assumed to have the same distribution as human BSEP reported in (Meier et al., 2006); similar expression ranges are also reported in (Bernhardt et al., 2012); all uptake Vmax values are covariant
BA_baso_Vmax	Bulk bile acid basolateral transport Vmax	All transporters were assumed to have the same distribution as human BSEP reported in (Meier et al., 2006); similar expression ranges are also reported in (Bernhardt et al., 2012); all basolateral Vmax values are covariant

BA_canal_Vmax	Bulk bile acid canalicular transport Vmax	All transporters were assumed to have the same distribution as human BSEP reported in (Meier et al., 2006); similar expression ranges are also reported in (Bernhardt et al., 2012); all canalicular Vmax values are covariant
BA_synthesis_So	Initial bulk bile acid synthesis rate	Used 50-fold lognormal range of variability in metabolizing enzymes in line with variability reported in (Tracy et al., 2016)
CDCA_synthesis_So	Initial CDCA synthesis rate	Used 50-fold lognormal range of variability in metabolizing enzymes in line with variability reported in (Tracy et al., 2016)
LCA_uptake_Vmax	LCA uptake Vmax	All transporters were assumed to have the same distribution as human BSEP reported in (Meier et al., 2006); similar expression ranges are also reported in (Bernhardt et al., 2012); all uptake Vmax values are covariant
LCA_baso_Vmax	LCA basolateral transport Vmax	All transporters were assumed to have the same distribution as human BSEP reported in (Meier et al., 2006); similar expression ranges are also reported in (Bernhardt et al., 2012); all basolateral Vmax values are covariant
LCA_canal_Vmax	LCA canalicular transport Vmax	All transporters were assumed to have the same distribution as human BSEP reported in (Meier et al., 2006); similar expression ranges are also reported in (Bernhardt et al., 2012); all canalicular Vmax values are covariant
LCAamide_uptake_Vmax	LCA-amide uptake Vmax	All transporters were assumed to have the same distribution as human BSEP reported in (Meier et al., 2006); similar expression ranges are also reported in (Bernhardt et al., 2012); all uptake Vmax values are covariant
LCAamide_baso_Vmax	LCA-amide basolateral transport Vmax	All transporters were assumed to have the same distribution as human BSEP reported in (Meier et al., 2006); similar expression ranges are also reported in (Bernhardt et al., 2012); all basolateral Vmax values are covariant
LCAamide_canal_Vmax	LCA-amide canalicular transport Vmax	All transporters were assumed to have the same distribution as human BSEP reported in (Meier et al., 2006); similar expression ranges are also reported in (Bernhardt et al., 2012); all canalicular Vmax values are covariant

LCAsulfate_uptake_Vmax	LCA-sulfate uptake Vmax	All transporters were assumed to have the same distribution as human BSEP reported in (Meier et al., 2006); similar expression ranges are also reported in (Bernhardt et al., 2012); all uptake Vmax values are covariant
LCAsulfate_baso_Vmax	LCA-sulfate basolateral transport Vmax	All transporters were assumed to have the same distribution as human BSEP reported in (Meier et al., 2006); similar expression ranges are also reported in (Bernhardt et al., 2012); all basolateral Vmax values are covariant
LCAsulfate_canal_Vmax	LCA-sulfate canalicular transport Vmax	All transporters were assumed to have the same distribution as human BSEP reported in (Meier et al., 2006); similar expression ranges are also reported in (Bernhardt et al., 2012); all canalicular Vmax values are covariant
CDCA_uptake_Vmax	CDCA uptake Vmax	All transporters were assumed to have the same distribution as human BSEP reported in (Meier et al., 2006); similar expression ranges are also reported in (Bernhardt et al., 2012); all uptake Vmax values are covariant
CDCA_baso_Vmax	CDCA basolateral transport Vmax	All transporters were assumed to have the same distribution as human BSEP reported in (Meier et al., 2006); similar expression ranges are also reported in (Bernhardt et al., 2012); all basolateral Vmax values are covariant
CDCA_canal_Vmax	CDCA canalicular transport Vmax	All transporters were assumed to have the same distribution as human BSEP reported in (Meier et al., 2006); similar expression ranges are also reported in (Bernhardt et al., 2012); all canalicular Vmax values are covariant
CDCAamide_uptake_Vmax	CDCA-amide uptake Vmax	All transporters were assumed to have the same distribution as human BSEP reported in (Meier et al., 2006); similar expression ranges are also reported in (Bernhardt et al., 2012); all uptake Vmax values are covariant
CDCAamide_baso_Vmax	CDCA-amide basolateral transport Vmax	All transporters were assumed to have the same distribution as human BSEP reported in (Meier et al., 2006); similar expression ranges are also reported in (Bernhardt et al., 2012); all basolateral Vmax values are covariant

CDCAamide_canal_Vmax	CDCA-amide canalicular transport Vmax	All transporters were assumed to have the same distribution as human BSEP reported in (Meier et al., 2006); similar expression ranges are also reported in (Bernhardt et al., 2012); all canalicular Vmax values are covariant
CDCA_amidation_Vmax	CDCA amidation Vmax	Used 50-fold lognormal range of variability in metabolizing enzymes in line with variability reported in (Tracy et al., 2016)
LCA_synthesis_Vmax	LCA synthesis Vmax	Assumed parameter range of ±2 orders of magnitude with ±50% standard deviation and validated with outcome data
LCAamide_sulfation_Vmax	LCA-amide sulfation Vmax	Used 50-fold lognormal range of variability in metabolizing enzymes in line with variability reported in (Tracy et al., 2016)
canal_reg_scale	Canalicular transporter regulation exponent	Assumed parameter range of 0-8 with ±50% standard deviation and validated with outcome data
baso_reg_scale	Basolateral transporter regulation exponent	Assumed parameter range of 0-8 with ±50% standard deviation and validated with outcome data
uptake_reg_scale	Uptake transporter regulation exponent	Assumed parameter range of 0-8 with ±50% standard deviation and validated with outcome data
Vmax_renal_BA	Renal bile acid excretion Vmax	Assumed to have same distribution as BSEP reported in (Meier et al., 2006)
Vmax_BA_gut	Bulk bile acid gut uptake Vmax	Range and distribution based on report in (Ho et al., 2011); all gut Vmax values treated as covariates
Vmax_CDCA_gut	CDCA gut uptake Vmax	Range and distribution based on report in (Ho et al., 2011); all gut Vmax values treated as covariates
Vmax_CDCAamide_gut	CDCA-amide gut uptake Vmax	Range and distribution based on report in (Ho et al., 2011); all gut Vmax values treated as covariates
Vmax_LCA_gut	LCA gut uptake Vmax	Range and distribution based on report in (Ho et al., 2011); all gut Vmax values treated as covariates
Vmax_LCAamide_gut	LCA-amide gut uptake Vmax	Range and distribution based on report in (Ho et al., 2011); all gut Vmax values treated as covariates
Vmax_LCAsulfate_gut	LCA-sulfate gut uptake Vmax	Range and distribution based on report in (Ho et al., 2011); all gut Vmax values treated as covariates

TABLE S2. Rat SimPops: Sprague-Dawley rat SimPops (Rat_ROS_apop_mito_BA_v8A_11, n=294)

Data used to Define Parameter Distributions (if applicable)		
**Parameter Symbol in DILIsym**	**Parameter Name in DILIsym**	**Data Source for Distribution**
ATP_decr_necrosis_Vmax	ATP decrement necrosis Vmax	Assumed standard deviation of ±20% and parameter range of 2.5 times the S.D. and validated with outcome data
Body_mass	Body Mass	Parameter range from (Solomon, 1977; Brown et al., 1997)
GSH_pre_trans_Vmax	GSH precursor transport Vmax	Parameter range from (Kim et al., 1992; Vendemiale et al., 1996; Chen et al., 2009)
GSHo	GSH basal level	Parameter range from (Lee et al., 2007; Nagasaka et al., 2009)
RNS_ROS_ATP_inhib_Vmax	RNS/ROS ATP inhibition Vmax	Parameter range from (Katyare and Satav, 1989)
RNS_ROS_cl_Vmax	Liver RNS/ROS baseline clearance Vmax	Assumed standard deviation of ±20% and parameter range of 2.5 times the S.D. and validated with outcome data
Basal_Stdzd_MitoETC_Flux	Basal value of mito ETC flux	Parameter range from (Flamment et al., 2009)
Resp_Reserve_Scalar	Scaling coefficient representing reserve mitochondria function	Parameter range from (Flamment et al., 2009)
CAS_apop_scale	Caspase-mediated apoptosis scaling constant	Parameter range derived from (Bantel et al., 2001)
HC_necr_HMGB1_release_rate	Released amount of HMGB1 by necrotic cells	Assumed standard deviation of ±20% and parameter range of 2.5 times the S.D. and validated with outcome data
TNF_mediated_necrosis_Vmax	TNF mediated necrosis Vmax	Assumed standard deviation of ±20% and parameter range of 2.5 times the S.D. and validated with outcome data
BA_uptake_Vmax	Bulk bile acid uptake Vmax	All transporters were assumed to have the same distribution as human BSEP reported in (Meier et al., 2006) due to lack of similar rat data; all uptake Vmax values are covariant
BA_baso_Vmax	Bulk bile acid basolateral transport Vmax	All transporters were assumed to have the same distribution as human BSEP reported in (Meier et al., 2006) due to lack of similar rat data; all basolateral efflux Vmax values are covariant
BA_canal_Vmax	Bulk bile acid canalicular transport Vmax	All transporters were assumed to have the same distribution as human BSEP reported in (Meier et al., 2006) due to lack of similar rat data

BA_synthesis_So	Initial bulk bile acid synthesis rate	Used 50-fold lognormal range of variability in metabolizing enzymes in line with variability reported in (Tracy et al., 2016)
CDCA_synthesis_So	Initial CDCA synthesis rate	Used 50-fold lognormal range of variability in metabolizing enzymes in line with variability reported in (Tracy et al., 2016)
LCA_uptake_Vmax	LCA uptake Vmax	All transporters were assumed to have the same distribution as human BSEP reported in (Meier et al., 2006) due to lack of similar rat data; all uptake Vmax values are covariant
LCA_baso_Vmax	LCA basolateral transport Vmax	All transporters were assumed to have the same distribution as human BSEP reported in (Meier et al., 2006) due to lack of similar rat data; all basolateral efflux Vmax values are covariant
LCA_canal_Vmax	LCA canalicular transport Vmax	All transporters were assumed to have the same distribution as human BSEP reported in (Meier et al., 2006) due to lack of similar rat data
LCAamide_uptake_Vmax	LCA-amide uptake Vmax	All transporters were assumed to have the same distribution as human BSEP reported in (Meier et al., 2006) due to lack of similar rat data; all uptake Vmax values are covariant
LCAamide_baso_Vmax	LCA-amide basolateral transport Vmax	All transporters were assumed to have the same distribution as human BSEP reported in (Meier et al., 2006) due to lack of similar rat data; all basolateral efflux Vmax values are covariant
LCAamide_canal_Vmax	LCA-amide canalicular transport Vmax	All transporters were assumed to have the same distribution as human BSEP reported in (Meier et al., 2006) due to lack of similar rat data; all canalicular efflux Vmax values are covariant
CDCA_uptake_Vmax	CDCA uptake Vmax	All transporters were assumed to have the same distribution as human BSEP reported in (Meier et al., 2006) due to lack of similar rat data; all uptake Vmax values are covariant

CDCA_baso_Vmax	CDCA basolateral transport Vmax	All transporters were assumed to have the same distribution as human BSEP reported in (Meier et al., 2006) due to lack of similar rat data; all basolateral efflux Vmax values are covariant
CDCA_canal_Vmax	CDCA canalicular transport Vmax	All transporters were assumed to have the same distribution as human BSEP reported in (Meier et al., 2006) due to lack of similar rat data; all canalicular efflux Vmax values are covariant
CDCAamide_uptake_Vmax	CDCA-amide uptake Vmax	All transporters were assumed to have the same distribution as human BSEP reported in (Meier et al., 2006) due to lack of similar rat data; all uptake Vmax values are covariant
CDCAamide_baso_Vmax	CDCA-amide basolateral transport Vmax	All transporters were assumed to have the same distribution as human BSEP reported in (Meier et al., 2006) due to lack of similar rat data; all basolateral efflux Vmax values are covariant
CDCAamide_canal_Vmax	CDCA-amide canalicular transport Vmax	All transporters were assumed to have the same distribution as human BSEP reported in (Meier et al., 2006) due to lack of similar rat data; all canalicular efflux Vmax values are covariant
CDCA_amidation_Vmax	CDCA amidation Vmax	Used 50-fold lognormal range of variability in metabolizing enzymes in line with variability reported in (Tracy et al., 2016)
LCA_synthesis_Vmax	LCA synthesis Vmax	Assumed parameter range of ±2 orders of magnitude with ±50% standard deviation and validated with outcome data
uptake_reg_scale	Uptake transporter regulation exponent	Assumed parameter range of 0-8 with ±50% standard deviation and validated with outcome data
baso_reg_scale	Basolateral transporter regulation exponent	Assumed parameter range of 0-8 with ±50% standard deviation and validated with outcome data
canal_reg_scale	Canalicular transporter regulation exponent	Assumed parameter range of 0-8 with ±50% standard deviation and validated with outcome data
Vmax_renal_BA	Renal bile acid excretion Vmax	Assumed to have same distribution as BSEP reported in (Meier et al., 2006)

Vmax_BA_gut	Bulk bile acid gut uptake Vmax	Range and distribution based on report in (Ho et al., 2011); all gut Vmax values treated as covariates
Vmax_CDCA_gut	CDCA gut uptake Vmax	Range and distribution based on report in (Ho et al., 2011); all gut Vmax values treated as covariates
Vmax_CDCAamide_gut	CDCA-amide gut uptake Vmax	Range and distribution based on report in (Ho et al., 2011); all gut Vmax values treated as covariates
Vmax_LCA_gut	LCA gut uptake Vmax	Range and distribution based on report in (Ho et al., 2011); all gut Vmax values treated as covariates
Vmax_LCAamide_gut	LCA-amide gut uptake Vmax	Range and distribution based on report in (Ho et al., 2011); all gut Vmax values treated as covariates
LCA_detox_Vmax	LCA detoxification Vmax	Given same range as transporters due to lack of quantitative data

Supporting Information 1 References

Allen, J.W., Shanker, G., and Aschner, M. (2001). Methylmercury inhibits the in vitro uptake of the glutathione precursor, cystine, in astrocytes, but not in neurons. Brain Res. *894*: 131–140.

Bantel, H., Ruck, P., Gregor, M., and Schulze-Osthoff, K. (2001). Detection of elevated caspase activation and early apoptosis in liver diseases. Eur. J. Cell Biol. *80*: 230–9.

Bernhardt, G. a, Zollner, G., Cerwenka, H., Kornprat, P., Fickert, P., Bacher, H., et al. (2012). Hepatobiliary transporter expression and post-operative jaundice in patients undergoing partial hepatectomy. Liver Int. Off. J. Int. Assoc. Study Liver *32*: 119–27.

Brown, R.P., Delp, M.D., Lindstedt, S.L., Rhomberg, L.R., and Beliles, R.P. (1997). Physiological parameter values for physiologically based pharmacokinetic models. Toxicol. Ind. Health *13*: 407–484.

Chen, Y.-H., Lin, F.-Y., Liu, P.-L., Huang, Y.-T., Chiu, J.-H., Chang, Y.-C., et al. (2009). Antioxidative and hepatoprotective effects of magnolol on acetaminophen-induced liver damage in rats. Arch. Pharm. Res. *32*: 221–228.

Flamment, M., Gueguen, N., Wetterwald, C., Simard, G., Malthièry, Y., and Ducluzeau, P.-H. (2009). Effects of the cannabinoid CB1 antagonist rimonabant on hepatic mitochondrial function in rats fed a high-fat diet. Am. J. Physiol. Endocrinol. Metab. *297*: E1162–70.

Ho, R.H., Leake, B.F., Urquhart, B.L., Gregor, J.C., Dawson, P.A., and Kim, R.B. (2011). Functional characterization of genetic variants in the apical sodium-dependent bile acid transporter (ASBT; SLC10A2). J. Gastroenterol. Hepatol. *26*: 1740–1748.

Katyare, S.S., and Satav, J.G. (1989). Impaired mitochondrial oxidative energy metabolism following paracetamol-induced hepatotoxicity in the rat. Br. J. Pharmacol. *96*: 51–58.

Kim, H.J., Rozman, P., Madhu, C., and Klaassen, C.D. (1992). Homeostasis of sulfate and 3’-phosphoadenosine 5’-phosphosulfate in rats after acetaminophen administration. J. Pharmacol. Exp. Ther. *261*: 1015–1021.

Lee, K.-T., Tsai, S.-M., Wang, S.-N., Lin, S.-K., Wu, S.-H., Chuang, S.-C., et al. (2007). Glutathione status in the blood and tissues of patients with virus-originated hepatocellular carcinoma. Clin. Biochem. *40*: 1157–1162.

Meier, Y., Pauli-Magnus, C., Zanger, U.M., Klein, K., Schaeffeler, E., Nussler, A.K., et al. (2006). Interindividual variability of canalicular ATP-binding-cassette (ABC)-transporter expression in human liver. Hepatol. Baltim. Md *44*: 62–74.

Nagasaka, H., Takayanagi, M., and Tsukahara, H. (2009). Children’s toxicology from bench to bed--Liver Injury (3): Oxidative stress and anti-oxidant systems in liver of patients with Wilson disease. J. Toxicol. Sci. *34 Suppl 2*: SP229-236.

Pérez-Carreras, M., Del Hoyo, P., Martín, M. a, Rubio, J.C., Martín, A., Castellano, G., et al. (2003). Defective hepatic mitochondrial respiratory chain in patients with nonalcoholic steatohepatitis. Hepatol. Baltim. Md *38*: 999–1007.

Shon, Y.-H., and Nam, K.-S. (2002). Protective effect of moutan cortex extract on acetaminophen-induced cytotoxicity in human Chang liver cells. Biol. Pharm. Bull. *25*: 1427–1431.

Solomon, S. (1977). Developmental changes in nephron number, proximal tubular length and superficial nephron glomerular filtration rate of rats. J. Physiol. 573–589.

Tracy, T.S., Chaudhry, A.S., Prasad, B., Thummel, K.E., Schuetz, E.G., Zhong, X.-B., et al. (2016). Interindividual Variability in Cytochrome P450-Mediated Drug Metabolism. Drug Metab. Dispos. Biol. Fate Chem. *44*: 343–351.

Vendemiale, G., Grattagliano, I., Altomare, E., Turturro, N., and Guerrieri, F. (1996). Effect of acetaminophen administration on hepatic glutathione compartmentation and mitochondrial energy metabolism in the rat. Biochem. Pharmacol. *52*: 1147–1154.
