# Supplementary material for: Quantitative systems toxicology (QST) reproduces species differences in PF‐04895162 liver safety due to combined mitochondrial and bile acid toxicity
Source: Pharmacol Res Perspect. 2019 Oct 9;7(6):e00523. doi: 10.1002/prp2.523 (PMC6785660; doi:10.1002/prp2.523)

Supporting Information 2

Supporting Information Results

In Vitro Experimental Data and Associated Parameter Values

TABLE S3. Nominal media concentrations (human and rat), measured media concentrations (human only), and measured cell-associated concentrations (human and rat) of PF-04895162 from primary hepatocytes (human or rat) cultured under the same conditions as used for the mitochondrial toxicity assays†

Human primary hepatocytes			Rat primary hepatocytes	
PF-04895162 nominal media conc. (μM)	PF-04895162 measured media conc.‡ (μM)	PF-04895162 cell associated conc.‡ (μM)	PF-04895162 nominal media conc. (μM)	PF-04895162 cell associated conc.‡ (μM)
1.2	0.5	9.6	1.2	51
3.7	1.2	28	3.7	102
11	2.9	95	11	353
33	6.2	368	33	1124
100	14	568	100	-§
300	47	-§	300	-§

† See Supplement 1 for culture conditions
‡ Actual media and cell-associated concentrations measured by mass spectrometry
§ Measurements could not be reliably determined

Assessing PF-04895162 induced Human Bile Acid Transporter Inhibition

The IC_{50} values of PF-04895162 for BA transporters were estimated from dose-response curves (Figure S1a-d). For MRP4, the IC_{50} value was directly calculated. For BSEP, MRP3, and NTCP, the IC_{50} value was estimated by extrapolation.

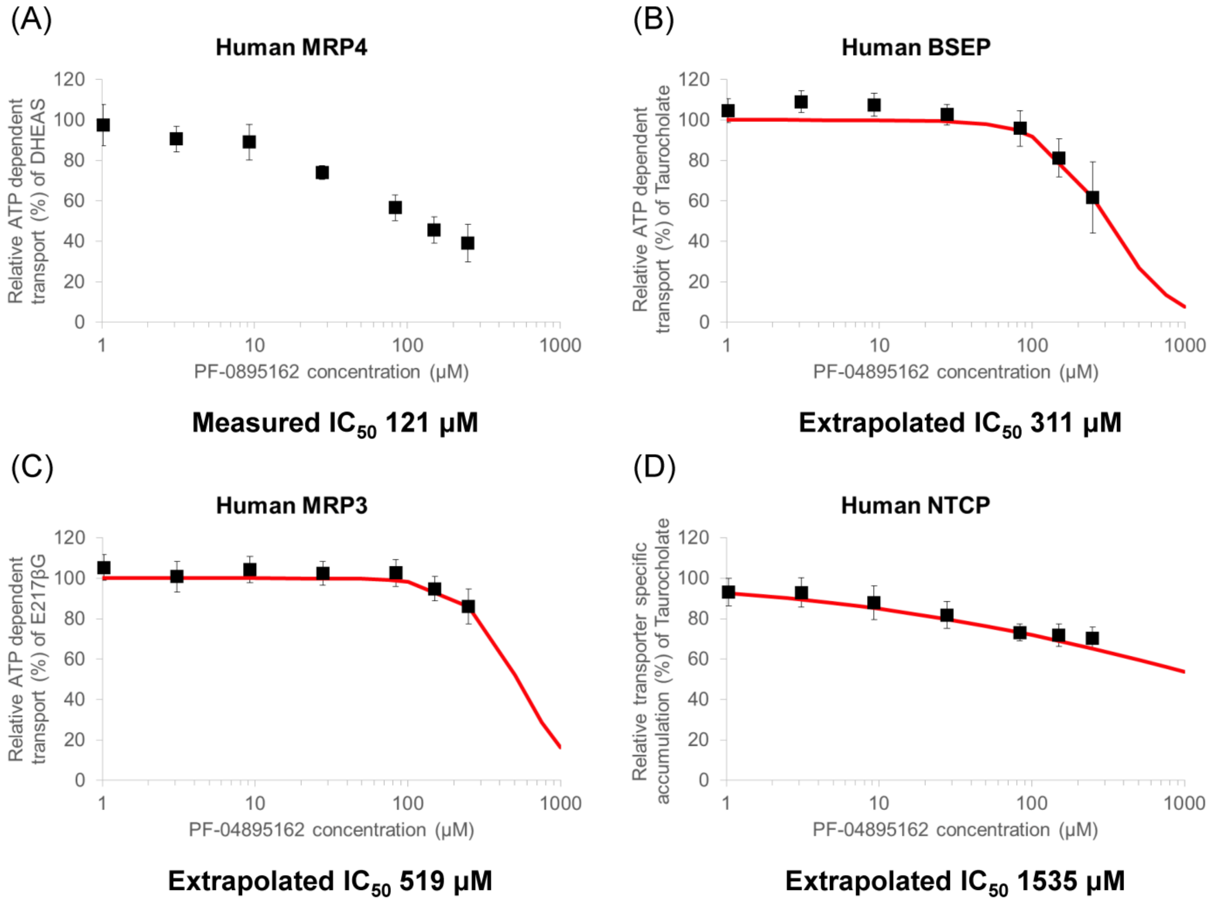

FIGURE S1. PF-04895162 induced inhibition of four major human bile acid transporters: (A) MRP4, (B) BSEP, (C) MRP3, and (D) NTCP. The IC_{50} values are indicated below their respective panels. The red line shows the estimated the best fit modeled using the expression: $\frac{100}{1+\left(\frac{IC_{50}}{[drug]}\right)^{n}}$.

Assessing PF-04895162 induced Rat Bile Acid Transporter Inhibition

The IC_{50} values of PF-04895162 for BA transporters were estimated from dose-response curves (Fig S2). For the rat transporters, IC_{50} values were directly calculated.

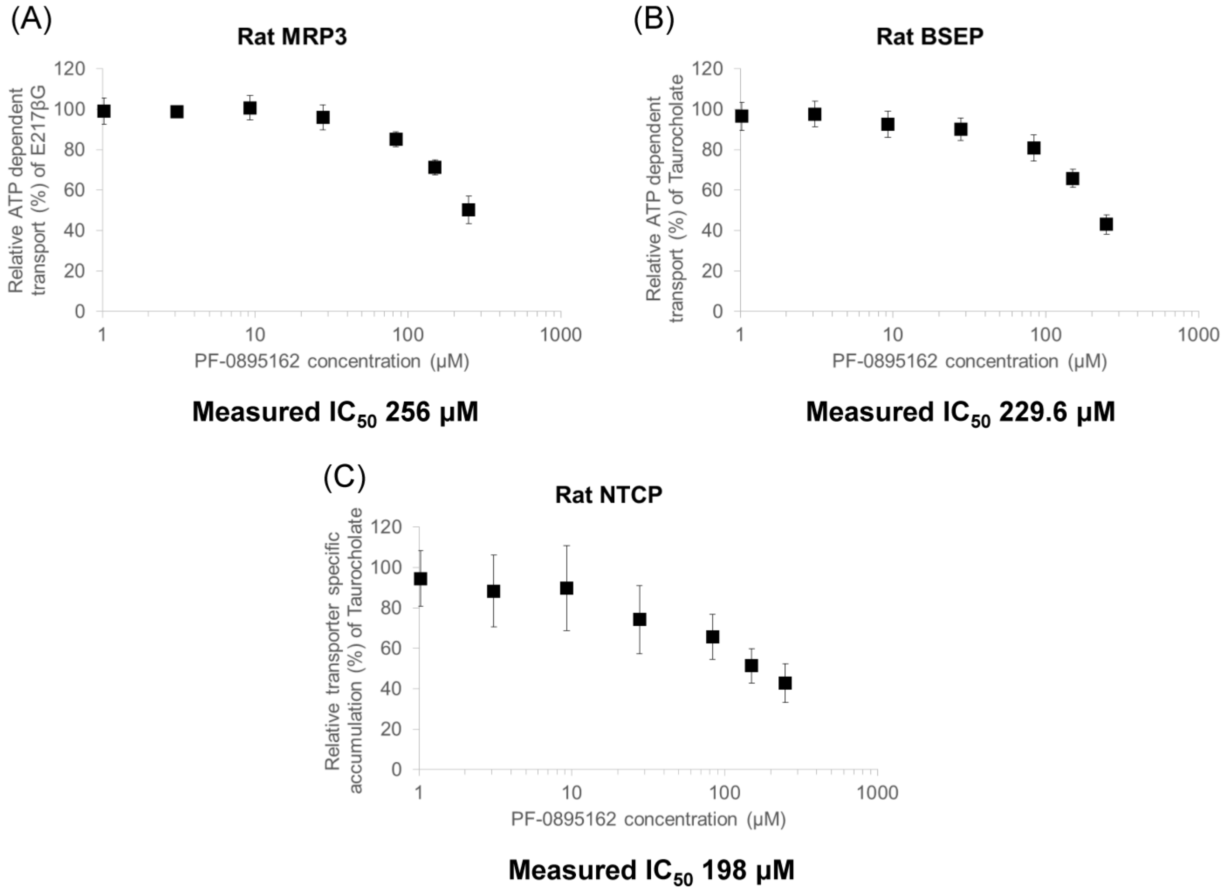

FIGURE S2. PF-04895162 induced inhibition of three major rat bile acid transporters (A) MRP3, (B) BSEP and (C) NTCP. All IC_{50} values could be calculated directly from the measured data and are indicated below their respective panels.

Assessing Human Mitochondrial Toxicity of PF-04895162

Human hepatocytes were incubated with varying concentrations of PF-04895162 for 24 hours. The effect of PF-04895162 on cellular respiration was assessed using a Seahorse XF Analyzer. Initially, the effect on basal respiration was assessed by measurement of oxygen consumption rate (OCR). Then the effect on spare respiratory capacity was assessed following addition of FCCP and measurement of a PF-04895162-mediated change in FCCP-stimulated OCR.

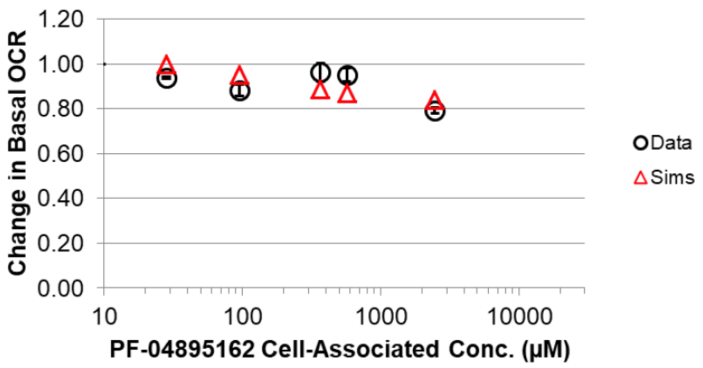

FIGURE S3. Dose-dependent effects of PF-04895162 on basal OCR were plotted relative to the measured cell-associated concentrations†. MITOsym was utilized to reproduce the Seahorse data by representing PF-04895162 as a mild ETC inhibitor, K_m 312 µM, V_{max} 0.414. †Measurement of cell-associated PF-04895162 in human hepatocyte cultures treated with 300 µM nominal media concentration were unreliable. Thus, the cell associated concentration corresponding to 300 µM nominal media concentration was calculated as 300 µM multiplied by (the average ratio between cell associated and nominal media concentrations for all concentrations).

Assessing Rat Mitochondrial Toxicity of PF-04895162

Similar to human hepatocytes, rat hepatocytes were incubated with varying concentrations of PF-04895162 for one or 24 hours. The effect of PF-04895162 on cellular respiration was assessed using a Seahorse XF Analyzer. Initially, the effect on basal respiration was assessed by measurement of oxygen consumption rate (OCR). Then the effect on spare respiratory capacity was assessed following addition of FCCP and measurement of a PF-04895162-mediated change in FCCP-stimulated OCR.

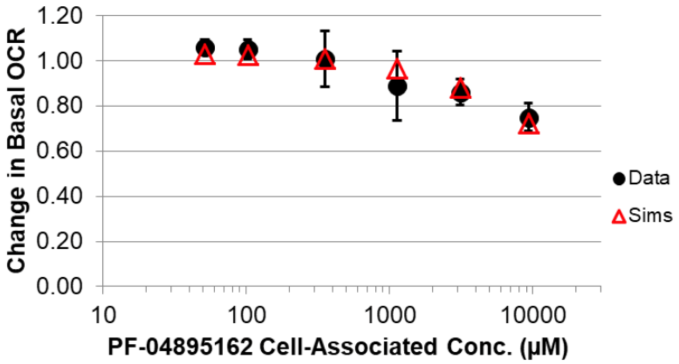

FIGURE S4. Dose-dependent effects of PF-04895162 on basal OCR were plotted relative to the measured cell-associated concentrations or a scaled cell-associated concentration if the measured data were unreliable. MITOsym was utilized to reproduce the Seahorse data by representing PF-04895162 as a mild ETC inhibitor, K_m 1500 µM.

Summary of PF-04895162 Mitochondrial and Bile Acid Toxicity Parameters in Rat and Human

TABLE S4. DILIsym toxicity parameter values. This table summarizes the toxicity parameters for PF-04895162. The IC_{50} values for the bile acid transporters can be directly applied to DILIsym. The mitochondrial toxicity parameters were first fit in MITOsym, then converted for use in DILIsym.

Parameter Name	Human Parameter Value	Rat Parameter Value	Units
**BA canalicular efflux inhibition constant†**	311	229.6	µM
**BA basolateral efflux inhibition constant†, ‡**	121	256	µM
**BA influx inhibition constant†**	1535	198	µM
**ETC3§ K_m**	312	-	µM
**ETC3§ V_{max}**	0.414	-	dimensionless
**ETC1§ K_m**	-	1500	µM

† The mode of inhibition is set to the default, mixed α=5

‡ Where MRP3 and MRP4 data are both available, the lower inhibition constant is used, *i.e.,* the more conservative value from a safety perspective

§ The appropriate ETC scaffold is selected based on the measured data. The ETC1 scaffold is relatively simple, using one parameter to characterize the sensitivity to inhibition by a compound. The ETC3 scaffold is more detailed, using two parameters that allow for a sustained response over a wide range of compound concentrations (*i.e.,* saturation).

PBPK Sub-Model Optimization

TABLE S5. This table summarizes the parameter values used to build the rat PBPK model within DILIsym. The Compound W scaffold was used.

DILIsym Parameter Name	Description	Value	Units
**Liver to blood**	Liver tissue to blood concentration ratio	5.74	dimensionless
**Muscle to blood**	Muscle tissue to blood concentration ratio	0.317	dimensionless
**Other tissue to blood**	"other" tissue to blood concentration ratio	0.1	dimensionless
**Fraction unbound**	Fraction of compound in plasma unbound to plasma proteins	0.019	dimensionless
**Molecular weight**	Molecular weight of compound	355.33	g/mol
**k(ab)**	Rate of absorption from the gut lumen to the gut tissue	0	1/hour
**Absorption from gut V_{max}**	Maximum rate of absorption from the gut lumen into the system	0.2	1/hour
**Absorption from gut K_m**	K_m for absorption from the gut lumen into the system	1.1	Mg
**Rate of elimination in feces**	First-order clearance from the gut lumen into the feces	0.15	1/hour
**K_m (metabolite A)**	Michaelis-Menten constant (K_m) for the conversion of parent to metabolite A	0.12	µmol/L
**V_{max} (metabolite A)**	Maximum reaction rate (V_{max}) for the conversion of parent to metabolite A	2640	nmol/hour/($kg^{0.75}$)

TABLE S6. This table summarizes the parameter values used to build the human PBPK model within DILIsym. The Compound Y scaffold was used.

DILIsym Parameter Name	Description	Value	Units
**k(ab,oral)**	Rate of absorption (ab) into the blood after an oral dose	0.5	1/hour
**Molecular Weight**	Molecular weight of compound	355.33	g/mol
**Hepatic Clearance**	Hepatic clearance rate	30000	mL/hour/($kg^{0.75}$)
**Oral Bioavailability**	Fraction of an oral dose that reaches the blood	0.75	dimensionless
**Volume of Distribution**	Volume of distribution	140	mL/kg
**Liver to Blood**	Liver tissue to blood concentration ratio	20	dimensionless
**Fraction Unbound Plasma**	Fraction of compound in plasma unbound to plasma proteins	0.004	dimensionless

FIGURE S5. Optimization simulations of PF-04895162 against data from rats administered a single dose of PF-04895162 (1mg/kg IV).

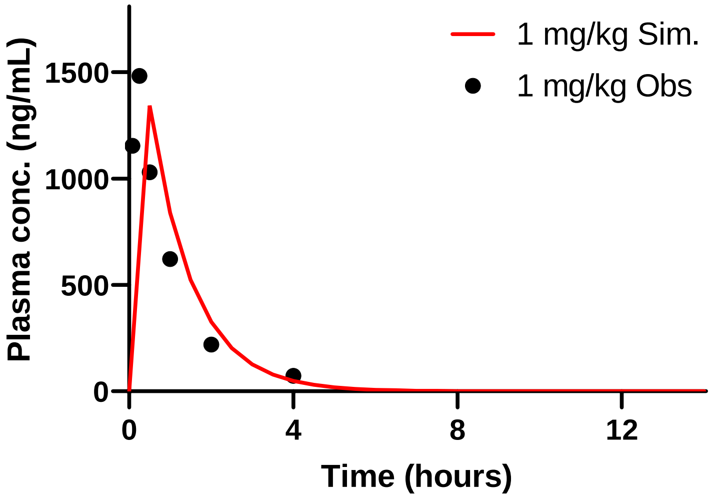

Additional Simulation Results

FIGURE S6. Optimization of an alternate rat PBPK sub-model, which prioritizes *in vitro* measurements of cell-associated PF-04895162 to inform the *in vivo* liver to blood partitioning. (A) Alternate optimization remains consistent with measured rat plasma PK data (B) Alternate optimization leads to higher simulated rat liver concentrations.

(A)

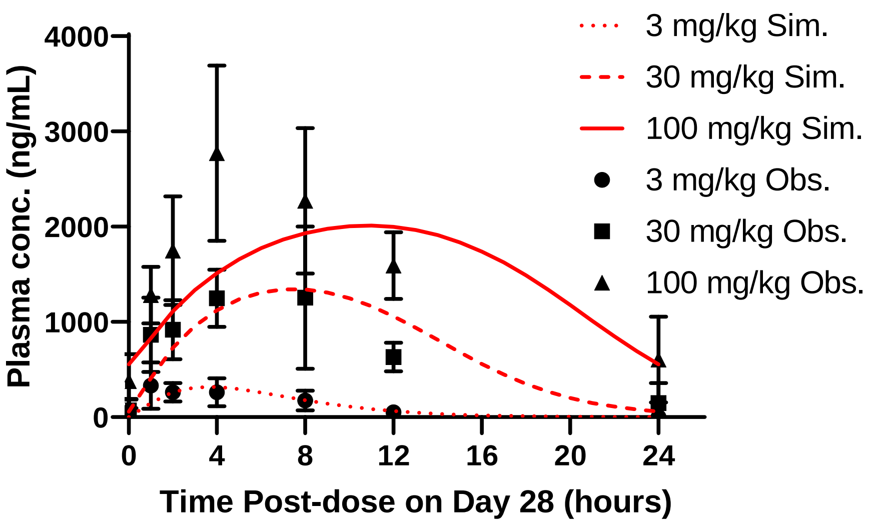

(B)

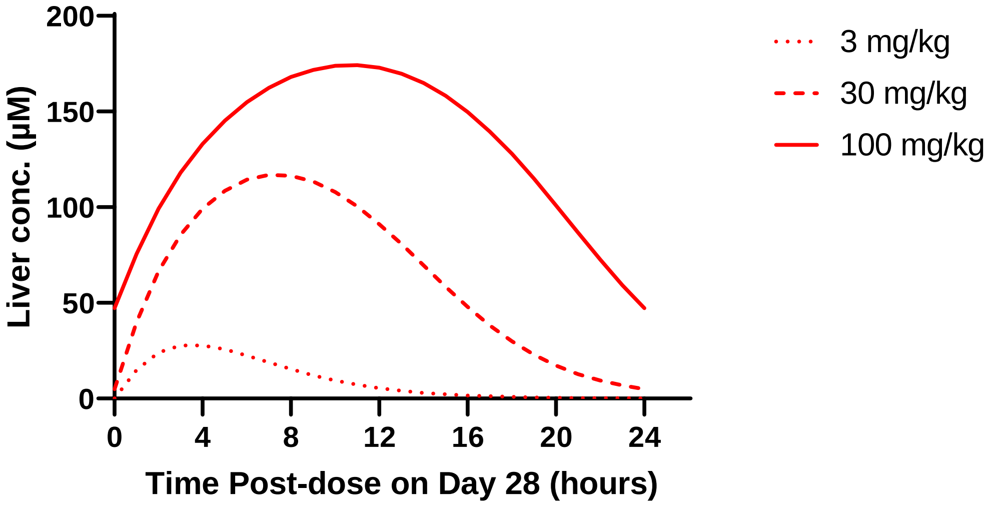

FIGURE S7. Simulation of PF-04895162 (100 mgkg-1day-1 for 28 days) in rat SimPops (n=294), using an alternate PBPK sub-model, remains negative for hepatotoxicity. Each star represents peak ALT and total bilirubin for an individual rat in the eDISH plot.

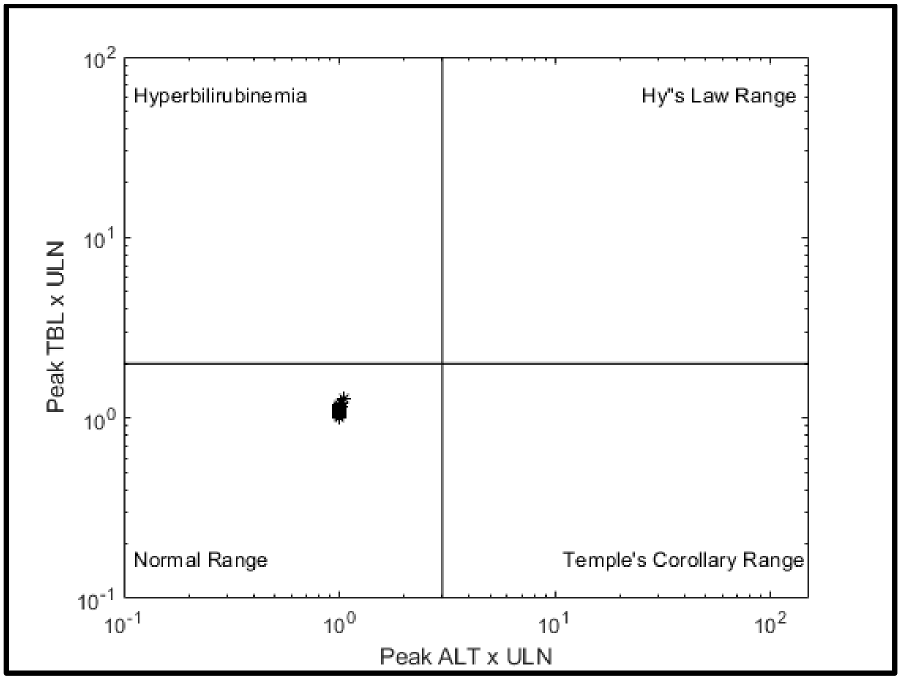

FIGURE S8. Subclinical simulated indicators of mechanisms of toxicity in rat SimPops, with adjusted PBPK representation leading to higher liver concentrations. (A) Liver CDCA-amide across the 28 day dosing period (100 mg/kg/day). (B) Liver average ATP across the 28 day dosing period (100 mg/kg/day). Each line represents an individual rat.

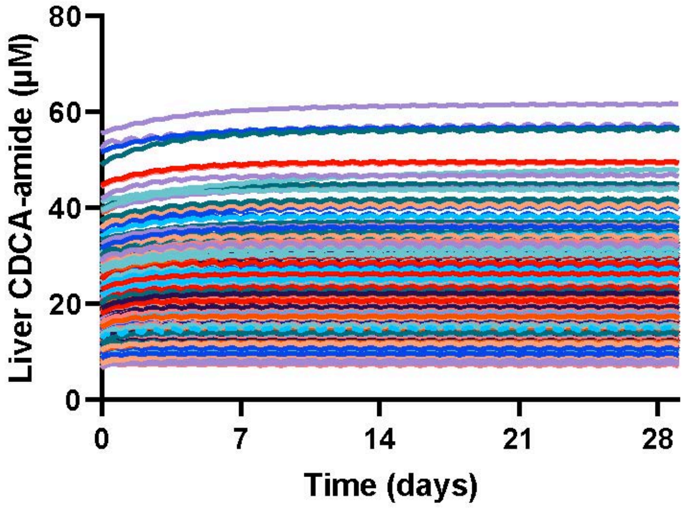

(A)

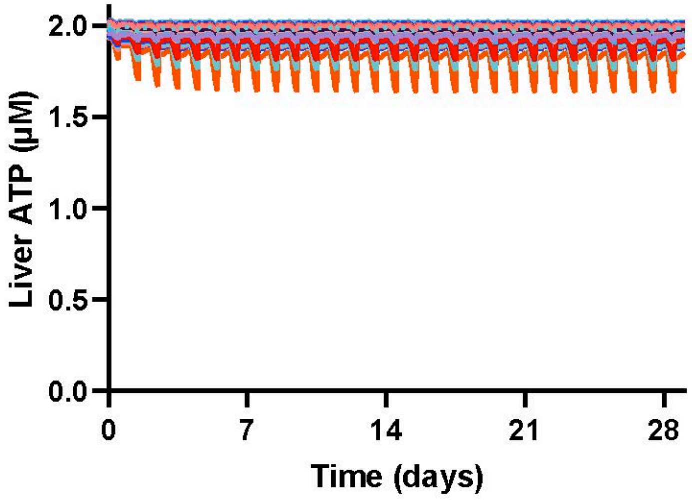

(B)

FIGURE S9. eDISH results of PF-04895162 (200mg BID, 7days) simulations in human SimPops (n=285), mirroring a clinical trial, shows only two responders (n=2) with peak ALT >3x ULN. Repeating these simulations with a slightly lower dose (170mg BID, 7days) shifts the two responders away from Temple's Corollary to the Normal Range; *i.e.* peak ALT <3x ULN, (results not shown). These two simulated patients had low body weights (50kg and 53kg), which may in part explain the discrepancy between the 200 mg BID simulations and the clinical trial, in which no patients (n=0/24) had ALT elevations greater than 3x ULN.

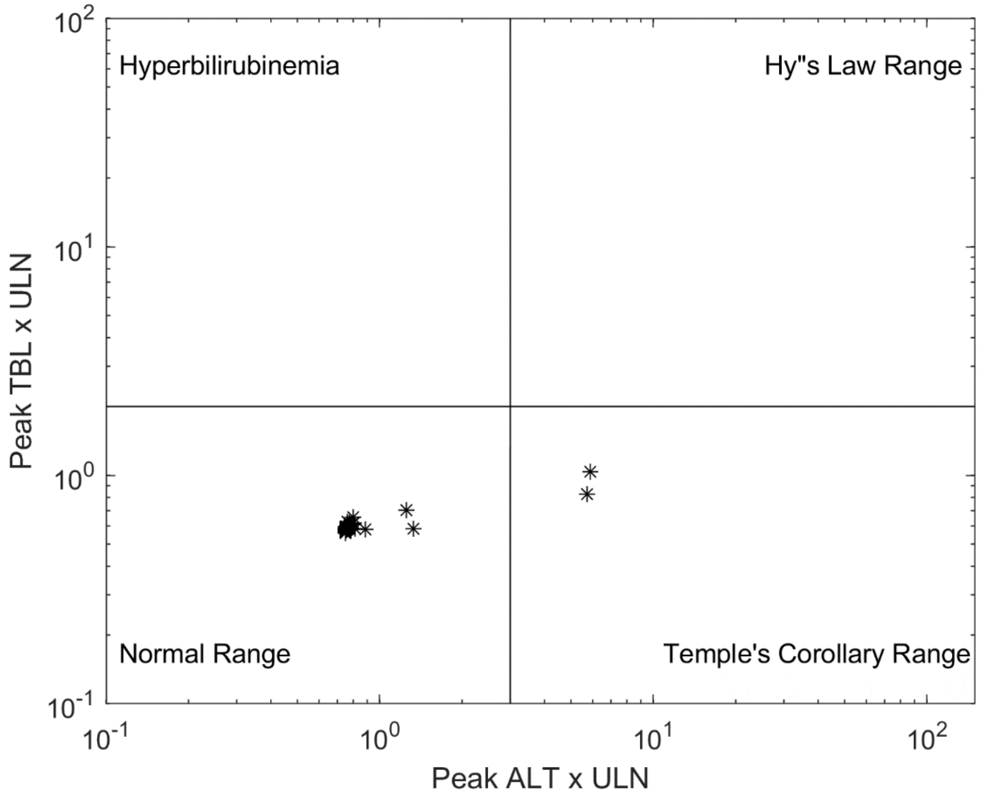

Supplement: Supplementary file 2 [file PRP2-7-e00523-s002.pdf]
